# Cellulose nanofibrils and silver nanoparticles enhances the mechanical and antimicrobial properties of polyvinyl alcohol nanocomposite film

**DOI:** 10.1038/s41598-022-23305-7

**Published:** 2022-11-08

**Authors:** Edwin Shigwenya Madivoli, Patrick Gachoki Kareru, Joyline Gichuki, Mostafa M. Elbagoury

**Affiliations:** 1 Chemistry Department, Jomo Kenyatta University of Agriculture and Technology, P.O Box 62, Nairobi, 000-00200 Kenya; 2 Faculty of Agriculture, Department of Biochemistry, AL-Azhar University, Cairo, Egypt

**Keywords:** Biopolymers, Nanocomposites, Polymer characterization, Polymer synthesis, Green chemistry, Sustainability, X-ray diffraction

## Abstract

Recent findings of microplastics in marine food such as fish, crabs and shrimps necessitate the need to develop biodegradable packaging materials. This study reports on the development of a biodegradable packing material from cellulose nanofibril-polyvinyl alcohol nanocomposite embedded with silver nanoparticles. Microcrystalline cellulose was isolated from sugarcane bagasse via the kraft process followed by conversion of cellulose I to cellulose II using NaOH/urea/water solution. The nanofibrils were then isolated using (2,2,6,6-Tetramethylpiperidin-1-yl) oxyl (TEMPO) and used as a reinforcing element in polyvinyl alcohol composite prepared through solvent casting. The tensile strength, water solubility, optical properties, water vapor permeability and wettability of the prepared films were then evaluated. The antimicrobial potency of the films was evaluated using the disc diffusion antimicrobial assay against selected microorganisms.

## Introduction

Escalated enquiries and disturbing reports have been stockpiling over the years concerning how unhealthy plastics are to the consumer more so with reference to food packaging^1,2^. Marine pollution by plastic debris^3,4^ and the infiltration of microplastics through the food web to cause hormonal imbalances, asthma, infertility, diabetes, cardiovascular disease and cancer are just but to sum up the magnitude of this problem^5–8^. A great urge in the scientific community has thereof been nurtured towards inaugurating bio-polymeric materials as sustainable substitutes to these toxic plastics^9^. This type of new packaging involves the use of natural sources or derivatives from organic monomers as raw materials^10^. Biodegradable polymers are classified depending on the raw materials used for their manufacture and the manufacturing processes and methods used to make them^11–13^. They could be obtained from biological sources such as animals, plants, agricultural remains and fossils or synthesized chemically from biopolymer monomeric units like sugars, amino acids etc^14,15^. Petroleum baseds biodegradable polymers such as polyvinyl alcohol (PVA), polyglycolic acid (PGA) and polylactic acid (PLA) are touted as vaible alternatives for use as packaging materials^15–17^.

Biopolymers, though highly biodegradable and a seemingly great substitute for petroleum based polymers, suffer several major drawbacks. Production of high value composites is hampered by their high processing costs and dismal performance though this is largely overcommed through polymer blending with nanoparticles or other biobased polymers^18,19^. This approach has been used to produce nanocomposites with enhanced physicochemmical properties such as environmental degradation, mechanical stability, UV shielding, and antimicrobial properties that leverages on the strength of each of the constituent materials^20–23^. Polymer blending has become one of the modern techniques for the development of new polymeric materials as it allows the manufacture of products with a larger scope of properties^24^. The biopolymer provides a compatible environment for the nanoparticles to utilise their high aspect ratio and surface area for maximum reinforcement^25,26^. PVA composites for various applications have greatly been explored with materials such as graphene, chitosan, cellulose etc^27–30^. This is because PVA has an opulence of surface hydroxyl groups making it highly hydrophilic, is non-toxic, has good biodegradability, provides a good framework for nano-composition and has great mechanical properties^31^. Cellulose alone, being insoluble in water, highly crystalline and fibrous would not be the best material for flexible film formation^32^. Therefore, cellulose nanofibrils (CNFs) have been used in combination with PVA in several studies to boost tensile strength, antimicrobial activity, swelling properties and biodegradability of these films^29,33–36^. The nanofibrils have high dispersibility in polymeric matrices, as they form strong hydrogen bonds with the hydroxyl groups of the polymer to synchronise a fortifying effect to the film^37^. CNFs have also been applied as anchoring sites for metal ions to serve as hubs for the nano-creation of metal nanoparticles^38^.

Cellulose-nano silver composite films are shown to be non-toxic, have strong antimicrobial activity and catalytic properties^39–41^. The incorporation of nanoparticles into food packaging has been beneficial because they endow various tailor-made properties in the manufacturing of polymeric films. Typically, polyolefin such as polyethylene, and biopolymers such as polylactic acid (PLA), are often fabricated in nanocomposite with engineered nanoparticles (ENPs) for functional food packaging. This is generally recognized as active packaging as the nanocomposite film can improve structural and thermal stability, endow gas/moisture barrier properties and strong antibacterial activity, ultimately extending the shelf life of the food. This study therefore, sought to prepare a composite film comprising tempo oxidized cellulose nanofibrils (TOCNF) and polyvinyl alcohol (PVA) impregnated with silver nanoparticles (AgNPs) that were generated in-situ. The films were prepared by solvent casting a solution of TOCNF, PVA and AgNPs in plastic moulds then evaporating off the liquid in an oven to give a dry nanocomposit material. The tensile strength, degree of elongation and Young’s modulus were used to evaluate the mechanical properties of the developed film while water vapor permeability, solubility, wettability and microbial inhibition were used to evaluate the films applicability as a packaging material.

## Experimental

### Preparation of hybrid AgNPs@PVA-CNF nanocomposite films

Raw bagasse was washed with distilled water to remove impurities and dried overnight in an oven set at 80 °C^42,43^. Cellulose was then obtained from the dried biomass through treating the dried biomass with 10% NaOH (1:10 w/v) at 80 °C for 3 h, washing and drying to constant weight. The alkali-treated biomass was subsequently treated twice with 30% H_2_O_2_ (1:6 w/v) with the addition of 50 ml 10% NaOH as a catalyst and left to react for 6 h for bleaching to occur. 1 g of cellulose was then dispersed in 10 ml NaOH/urea/water solution (10:10:80 by volume) then frozen at − 10 °C for 24 h^44^. After 24 h, the resultant cellulose solution thawed to obtain a transparent solution which was then coagulated with acetic acid (5 ml dope:1 ml acid), washed with distilled water and oven-dried to obtain cellulose II^41,45^. To obtain CNFs, the 20 g of cellulose were dispersed in 200 ml of water in which 6.4 mmol of TEMPO and 97 mmol of NaBr had previously been dissolved in it. The oxidation was initiated by dropwise addition of 50 ml of 14 vol.% sodium hypochlorite (NaOCl) solution for 10 min at room temperature and the reaction was allowed to progress for 3 h while maintaining the pH at 10 by periodic addition of 0.5 M NaOH solution. After 3 h, the reaction was quenched by addition of ethanol, washed through centrifugation and dried in an oven set at 80 °C^41,46–48^. Hybrid AgNPs@PVA-CNFs were prepared by dispersing 0.2 g of CNF and 1 g of Polyvinyl alcohol (PVA) in 50 ml of 100 mM AgNO_3_ solution. The reaction vessel was then wrapped with aluminium foil and heated at 80 °C for 4 h to allow for the hydrothermal reduction of silver ion to silver nanoparticles in PVA-CNF^41,49,50^. Pure PVA films without AgNPs@CNFs were also prepared as a control.

### FT-IR functional group analysis

The functional groups present in the AgNPs@PVA-CNF nanocomposites were determined using a Shimadzu FT-IR spectrophotometer (Shimadzu, Japan). The KBr pellets of the samples were prepared by grinding 10 mg of samples with 250 mg KBr (FT-IR grade). The 13 mm KBr pellets were prepared in a standard device under a pressure of 75 kN cm^−2^ for 3 min. The spectral resolution was set at 4 cm^−1^ and the scanning range from 400 to 4000 cm^−1^^41,51–53^.

### Optical properties of the composite films

To study the optical characteristics of the composite films, a Shimadzu UV–Vis 1800 spectrophotometer (Shimadzu, Japan) was used to evaluate the amount of light transmitted by the films. The transparency of the films and their UV-shielding abilities were determined by calculating their percent transmittance of light at 280 and 660 nm, respectively^54^.

### Thickness of the film

The thickness of the films was measured using a micrometre screw gauge to the nearest 0.001 mm. Measurements were made in at least seven random locations of each preconditioned film and the values were reported as mean ± standard deviation (SD). The mean values were used to determine the tensile strength of the dried films^55–58^.

### Tensile strength

The tensile strength was measured using an in-house fabricated tensile strength device constructed from a laboratory clamp and stand and metal suspensions. The rectangular-cut (10 × 50 mm) film specimens were sandwiched on both ends between two small metal brackets which were then clamped to a slotted mass hanger where successive 5 g increments of weights were added. The extension lengths were recorded after each addition and the total weight at film breakage was recorded. The tensile strength was calculated by dividing the total load at the breakpoint (in newtons) by the original cross-sectional area of the segment of the specimen that broke (in square meters) according to the following equation^58,59^

1 $$\mathrm{ts}= \frac{\mathrm{W }\left(\mathrm{kg}\right).\left(9.80 \frac{\mathrm{N}}{\mathrm{kg}}\right)}{\mathrm{A }\left({10}^{-4}\frac{{\mathrm{m}}^{2}}{{\mathrm{cm}}^{2}}\right)},$$

where W = total load, A = cross sectional area of the film.

The percent elasticity (% E) was calculated using the following equation:

2 $$\mathrm{\%E}= \frac{{\mathrm{L}}_{\mathrm{B}}}{{\mathrm{L}}_{\mathrm{I}}}\mathrm{ x }100,$$

where L_B_ and L_I_ are length at break and initial length of the films respectively. The elastic modulus (E) was determined from the slope of the stress–strain curve^60^.

### Water vapor permeability, wettability and degree of solubility

To determine the films’ solubility and swelling capacity, film pieces (20 × 20 mm) were dried at 105 °C to constant weight followed by immersion in 50 mL of distilled water and shaken gently for 24 h at 25 °C. The solutions were poured onto a filter paper (Whatman #1) to recover the undissolved films which were then weighed, and the wettability calculated using Eq. (3):

3 $$\mathrm{Swelling }\left(\mathrm{\%}\right)=\frac{\mathrm{Final\,wet\,weight}-\mathrm{initial\,dry\,mass}}{\mathrm{Initial\,dry\,mass}}\mathrm{ x }100$$

The film solubility was determined as the percentage dry matter of the remaining film after immersion in water. Film pieces (20 × 20 mm) were dried at 105 °C to constant weight followed by immersion in 50 mL of distilled water and shaken gently for 24 h at 25 °C. The solutions were poured onto a filter paper (Whatman #1) to recover the undissolved films. The samples were then rinsed with distilled water and dried at 105 °C to constant weight and the solubility was calculated according to Eq. (4) ^55,57^.

4 $$\mathrm{Solubility }\left(\mathrm{\%}\right)=\frac{\mathrm{Initial\,dry\,mass}-\mathrm{final\,dry\,mass}}{\mathrm{Initial\,dry\,mass}}\mathrm{ x }100$$

To determine the water vapor transmission rate, 40 mL of distilled water was poured into a beaker which was then covered with the film and air tightened by adhesive tape to prevent the escape of vapour as the beaker was heated. The beaker was weighed (W_i_) and placed in an oven at 50 °C for 24 h and then re-weighed again (W_f_). The WVTR (g/m^2^h) was then calculated using the Eq. (5):

5 $$\mathrm{WVTR}= \frac{{\mathrm{W}}_{\mathrm{i}}-{\mathrm{W}}_{\mathrm{f}}}{\mathrm{A}}\mathrm{ x }24$$

where W_i_ and W_f_ are the initial and final weight of the beaker (g) respectively and A is the cross-sectional area of the beaker (m^2^)^60,61^.

### Disk diffusion antimicrobial tests

The antimicrobial activity of the prepared films was evaluated using five different pathogenic microbes as test strains: *Escherichia coli* (Ec), *Bacillus subtilis* (Bs), *Staphylococcus aureus* (Sa)*, Pseudomonas aeruginosa* (Pa) and *Candida albicans* (Ca) by an inhibition zone assay method^41,62^. To prepare the agar diffusion assay 20 mL of 38 g/L solution of Mueller Hinton Agar was autoclaved at 121 °C for 15 min and cooled to 45 °C. Using a sterile pipette, 100 µL of the bacterial broth culture of the test organism was inoculated with 10 mL aliquots of nutrient broth, spread evenly with a sterile spreader onto sterile petri dishes to get a bacterial lawn and incubated at 37 °C for 24 h. The test circular films with a diameter of 0.5 cm were cut and placed in triplicate on the surface of the agar plates. After 24 h incubation at 37 °C, the inhibition zone diameters were measured using a vaneer caliper to the nearest millimetre in comparison to inhibition diameters of vancomayacin and reported as mean ± standard deviations. Pure PVA film was used as the control^41,63^.

## Results and discussion

### Effect of reaction parameters on AgNPs synthesis

**Figure 1:**
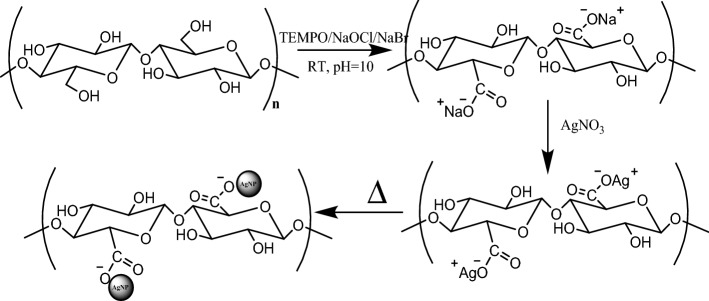
Schematic illustration showing the substitution of Ag^+^ ions onto PVA-TOCNFs, and the subsequent formation of AgNPs@PVA-TOCNFs.

**Figure 2:**
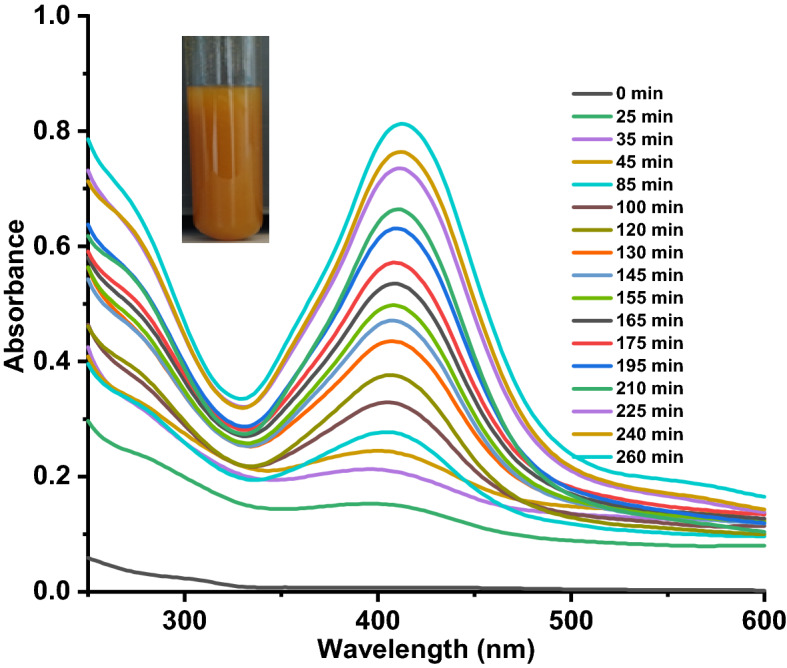
Changes in the surface plasmon reasonance peak (SPR) during the formation of AgNPs@CNFs.

In situ synthesis of AgNPs within cellulose nanofibrils matrix was followed through measurement of its plasmon resonance peak as shown in Figs. 1,2.

**Figure 3:**
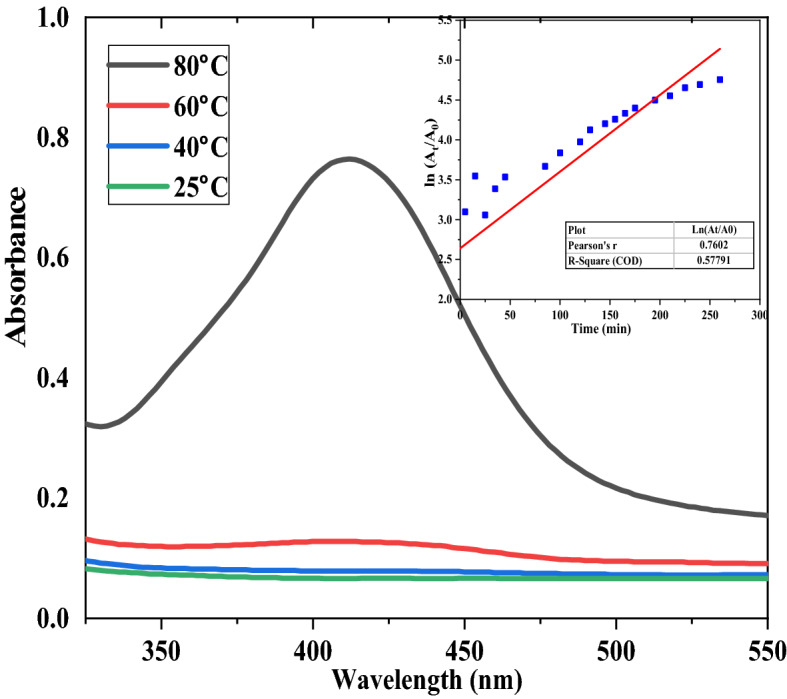
Effect of Temperature on the formation of AgNPs (**A**). Inset First order plot (**B**) of formation of AgNPs.

As shown in Fig. 1, when the CNFs were introduced to the AgNO_3_ solution, the Ag^+^ ions were paired by ion exchange to the negatively charged carboxylates on the CNF surface. These silver ions were subsequently reduced to silver nanoparticles in a temperature-dependent reaction that was also monitored using UV–Vis spectroscopy (Fig. 2)^64^. During the reduction of silver ions with CNFs, the colour of the solution turned from colourless to dark reddish-brown as the reaction progressed with a subsequent formation of an absorption peak at 412 nm. This peak has been associated with the plasmon resonance peak of AgNPs in solution and is the first qualitative indicator of the presence of the nanoparticles in solution^65^. At the beginning, the broader peak was due to fewer AgNPs formed after the conversion of a few Ag^+^ to Ag^0^ but as the reaction progressed, the intensity and height of the SPR peak increased over time since more Ag^0^ are being synthesized^65^. Figure 2 shows the final colour of the AgNPs suspension which turned from colourless to a weakly yellow colour, then to dark reddish-brown after a while. This is a clear indication of the formation of AgNPs. This brown colour change is very important during film casting since it acts as a shield for UV radiation^66^. Colour uniformity of the film is an indication of an even distribution of the AgNPs. TOCNFs can absorb di/trivalent metal cations due to the introduction of carboxyl groups at C6 position of TOCNF immobilise the AgNPs^49,67^. In comparison, the blank (pure AgNO_3_ solution) showed no spectral change in the λ_max_ or peak intensity over time. This is the SPR peak^66^, slightly shifting position as more AgNPs are agglomerated and which is the most favourable spectroscopic indicator for the formation of silver nanoparticles^68^. Figure 3 illustrates the effect of temperature variation and first-order plot of the formation of AgNPs 5 h of reaction time.

There was no significant change in the SPR peaks for temperatures 23 °C and 40 °C. A slight and very faint peak was seen after a while for the 60 °C solution. In comparison to the other tests, AgNPs prepared at 80 °C agglomerated significantly fast, just as also recorded by^68^. This shows that the preparation temperature for AgNPs plays an important role in their formation. Reaction time is also an important parameter. AgNPs began to form with an increasing concentration within the first 20 min and proceeded on steadily until the last minute. The increase in absorbance can be explained by the increase in the amount of the absorbing species (AgNPs)^69,70^ and from the data obtained it was found that the rate of AgNPs formation followed first-order kinetics given the high R-value obtained as shown in Fig. 3b. While some might argue that the presence of AgNPs in food packaging material poses a greater risk due to leaching of the nanoparticles, AgNPs antimicrobial packaging material are a promising form of active food packing^71^. To this end, the addition of AgNPs in polymeric matrices can influence the film permeability which subsequently influences product quality as it prevents the growth of food microorganisms such as *E. coli* and *S. aureus*^71^.

### Functional groups present in films

**Figure 4:**
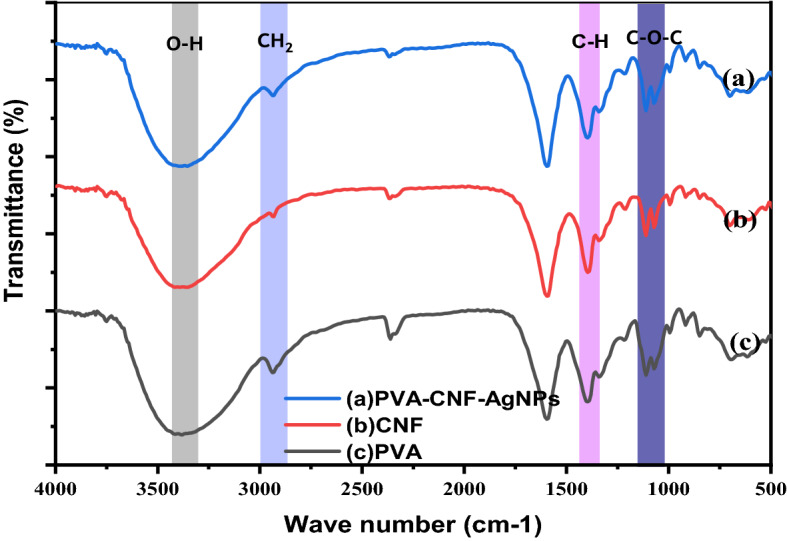
FTIR spectra of the PVA-based films (**a**) Ag-PT, (**b**) PVA-TOCNF, (**c**) Pure PVA.

Infrared spectroscopy was used to examine the nano-filling effects of AgNPs onto the PVA film and the results are depicted in Fig. 4.

The wide absorption peaks around 3394 cm^−1^ represent the stretching vibrations of hydrogen-bonded (− OH) groups of PVA, TOCNF, PVA-CNF-AgNPs^60^. This suggests good hydrophilicity of the films as the films can form hydrogen bonds with water^72^. From IR specta of PVA vibrational bands located at 2937, 1592, 1394, and 1068 cm^−1^ were associated with CH_2_ bending, OH vibrations from adsorbed water, CH_2_ stretching, and C–O–C vibrational frequencies^73^. On the other hand, TOCNF and nanocomposite spectra had vibrational bands at 1592, 1413, 1075 and 915 cm^−1^ associated with C = O, symmetric CH_2_ bending, C–O stretching and CH_2_ rocking. It should be noted that while PVA had a vibrational band at 1597 region, this band could not be linked to C = O band since pure PVA does not have carbonyl functional groups as compared to TOCNF and films which do have this group^74^. In the presence of metallic species, it has been reported that the vibrational band of C = O present in COOH shifts due to formation of carboxylate group containing metal ions. A weak band at 2943 cm^−1^ characteristic for C-H stretching^75^ is common to all films. Additional characteristic absorption bands at 1400 and 1075 cm^−1^ are also of C–H and O–H bending vibrations, respectively^41,65^.

### Tensile strength and transmittance of composite films

**Figure 5:**
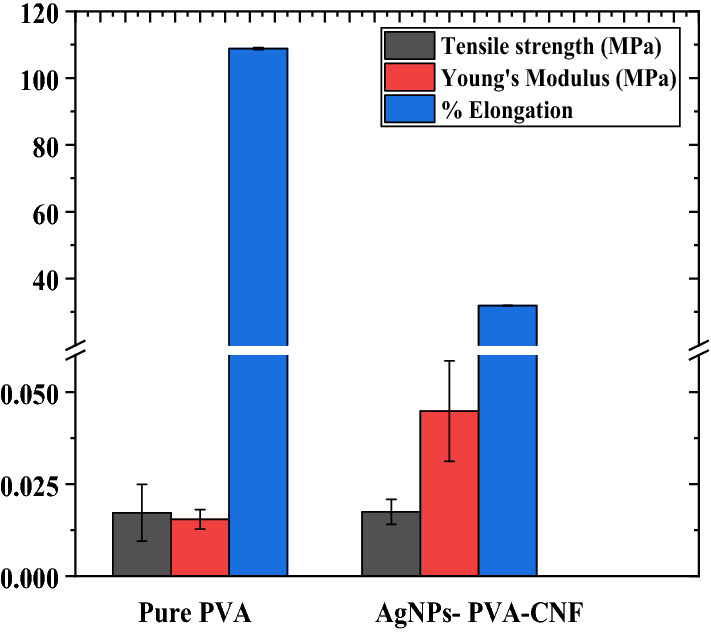
Comparison of the mechanical properties of the pure PVA and AgNPs@PVA-CNF composite films.

**Figure 6:**
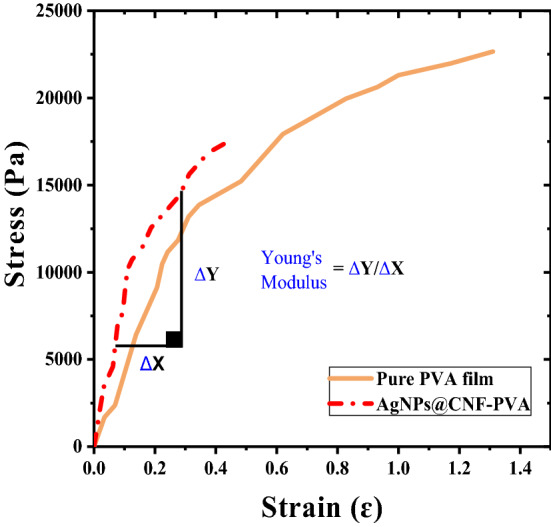
Stress and strain curves of PVA and AgNPs@CNF-PVA composite films.

Figures 5 and 6 show the mechanical properties of the two prepared films.

From the results, the tensile strength was increased from 1666 to 4001.7 Pa when CNF and AgNPs were incorporated in PVA matrix. This shows that blending AgNPs-TOCNF into the PVA matrix produces stronger interactions between the nanofiller and polymer matrix which limits the matrix motion^49^. While this is case, percent elongation on the other hand decreased when the filler materials were added to the matrix which is an indication of increased flexibility of the films. On the other hand, the optical transmittance of the composite films was lower as compared to pure PVA films. Pure PVA films had a transmittance of 66.5% while AgNPs@CNF-PVA had a percent transmittance of 9.51 at 600 nm. Light-induced reaction led to the destruction of chlorophyll which leads to bleaching of certain vegetables, destruction of riboflavin in milk, oxidation of vitamin C, oxidation of carotenoid pigments and discoloration of fresh meat^76^. Hence the use of packing material that prevents exposure to light prevents food spoilage resulting from light-induced chemical spoilage. Thus, the low transmittance of AgNPs@CNF-PVA prevents exposure of food to light and in the long run reduces food spoilage because of light-induced chemical reactions^76^.

### Water vapour permeability (WVP), wettability (W) and solubility (S)

**Figure 7:**
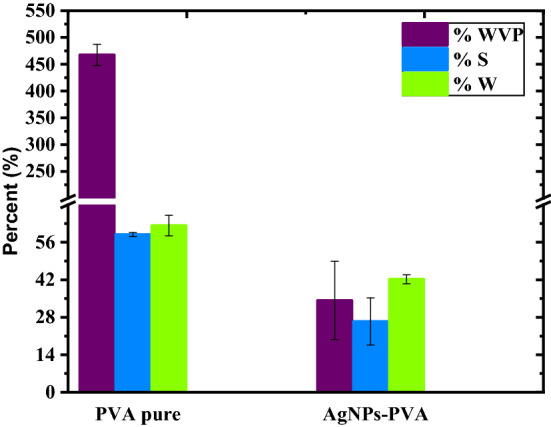
Moisture content, water vapor permeability, and water contact angle of agar and agar/cellulose composite film.

Figure 7 shows WVP, WS, and wettability values of the prepared films.

WVP is an important property in food packaging applications. Packaging material is the barrier between the exterior surrounding atmosphere and the packaged material inside^74^. The film is required to reduce to the minimum the moisture transfer from the outer surrounding, which would tamper with the freshness of the packaged food. In this study, pure PVA film had the very highest value: 467.49 g/smPa, whereas AgNPs-TOCNFs/PVA blended film had a very low value: 34.27 g/smPa. PVA is very hydrophilic making the film very unstable in aqueous solutions. This means more water vapour easily passes through the pure PVA film^60^. The TOCNFs in the composite film acts as an impermeable nanofiller to the PVA making the film more tortuous. It also complexes the molecular structure of the PVA providing the -COOH groups to bind to the − OH groups of the PVA thus reducing their hydrophilicity^77^. Extra moisture in the food allows for the development of microorganisms which in turn will render the food unsuitable for consumption after a very short span. With a very low WVP value, a film can extend food shelf-life up to considerable periods. As shown in Fig. 7, PVA is very soluble in water giving water solubility values of up to 60%. Upon blending the film with AgNPs-TOCNFs the film achieves more stability in water as the value considerably goes down to 26%. Several extrinsic factors such as temperature, humidity, oxygen, and water vapour have been reported as contributing factors to food spoilage during storage as bacterial growth is preferably favoured in moist conditions^76^, making the food harmful for human consumption^71^.

### X-ray diffractograms

**Figure 8:**
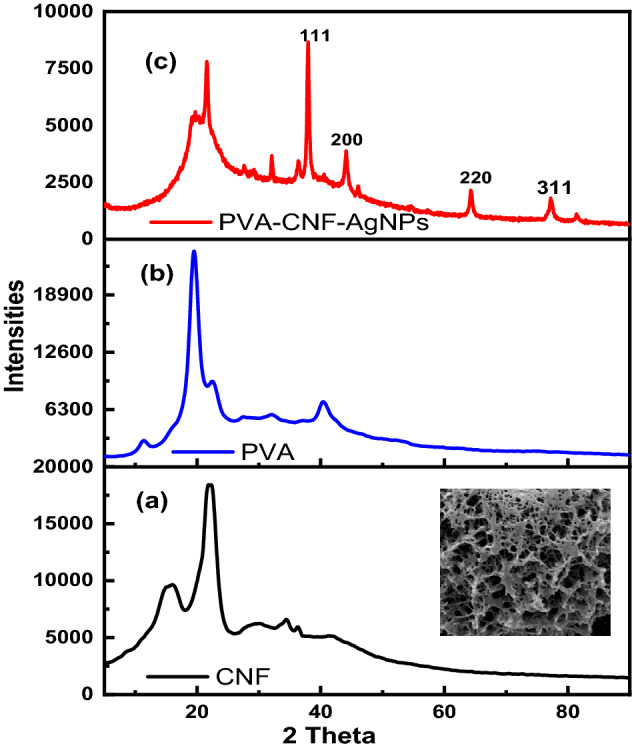
X-ray diffractograms of (**a**) CNF, (**b**) PVA and (**c**) AgNPs@PVA-CNF composite films.

Figure 8 depicts X-ray diffractograms of CNF, PVA and AgNPs@PVA-CNF.

Figure 8 shows the XRD patterns and corresponding crystallinity index (CrI) of CNF, PVA and AgNPs@PVA-CNF. Cellulose is composed of both crystalline and amorphous domains which tend to influence the XRD diffractogram observed^41,43,78^. The diffractogram of cellulose isolated from bagasse composed of a peaks at at 2 θ angles of 16°, 22°, 34°, respectively, and were attributed to the diffraction planes of (101), (002) and (040) crystalline plane cellulose ^41,43,78^. The diffractograms of PVA on the other, had one sharp peak with a shoulder at 2 θ = 19° and another small peak at 2 θ = 40° which upon incorporation of CNF within its marix led to overlapping of CNF and PVA peak that resulted in a broad peak centred at 2 θ = 20°. Sharp crystalline reflections, with a strong maximum at d = 4.68 Å (2 θ = 19.4°) and a shoulder at d = 4.43 Å (2θ = 20°), typical of the crystalline atactic PVA and they correspond to 101̄ and 101 reflections, respectively^79^. With respect to AgNPs@PVA-CNF, sharph distinct peaks at 2θ = 38, 44, 64, 77, and 82° associated with 111, 200, 220, 311 diffraction planes of silver nanoparticles respectively.

### TGA thermograms of nanocomposite films

**Figure 9:**
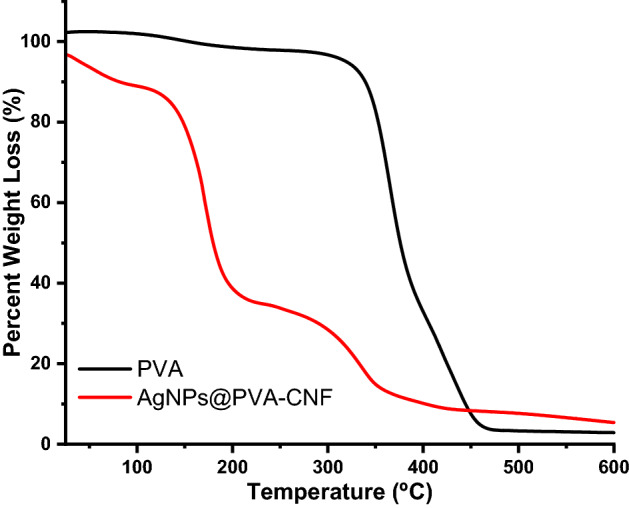
TGA thermograms of PVA and AgNPs@PVA-CNF composite films.

**Figure 10:**
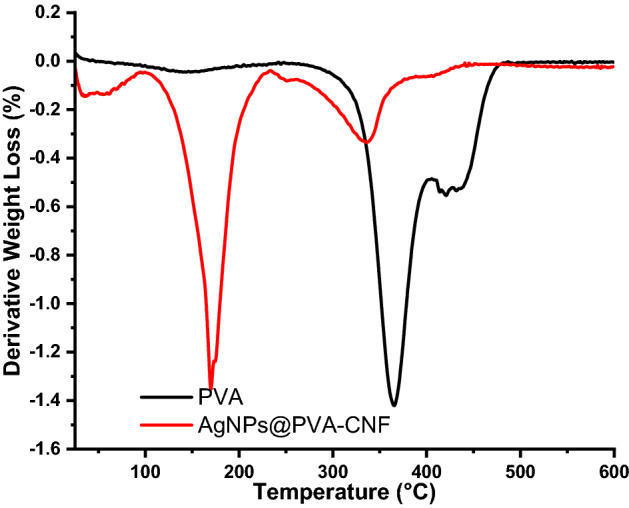
TGA thermograms of PVA and AgNPs@PVA-CNF composite films.

The thermal profile of PVA before and after incorporation of CNF and AgNPs were evaluated and the results are depicted in Figs. 9,10.

As can be seen from Figs. 9,10, the degradation temperature of the neat PVA films was 365 °C. On the other hand, the degradation temperature shifted to 170 °C when AgNPs@CNF was incorporated within its matrix. This shift in the degradation temperature was attributed to presence of CNF and AgNPs within the composite matrix. It should be noted that CNF has been shown to have two degradation cycles with the first one occurring between 50 and 100 °C attributed to adsorbed water while the second degradation cycle occurring between 220 and 380 °C attributed to breakdown of glucose chains^41,80^. In this study, the composite films had three degradation points at 45, 170 and 336 °C attributed to adsorbed water, breakdown of glucose units in cellulose and characteristic degradation of PVA via dehydration (or elimination of water from the PVA molecules), which resulted in the formation of polyene inter-mediate^41,80^. However, while it has been reported that strong hydrogen bonding interaction between the TOCNs and PVA matrix increase the thermal stability of the resultant composite films, this was not the case in this study. Here, presence of AgNPs within the matrix seemed to have played a role in the reduction of the thermal stability as the degradation temperature of the resultant film were lower than those observed in the neat films. It has been observed that that the disruption of hydrogen bond interactions due to the association of AgNPs with the COOH group introduced at C6 leads to the decrease in thermal stability of AgNPs@CNF^41,80^.

### SEM micrographs

**Figure 11:**
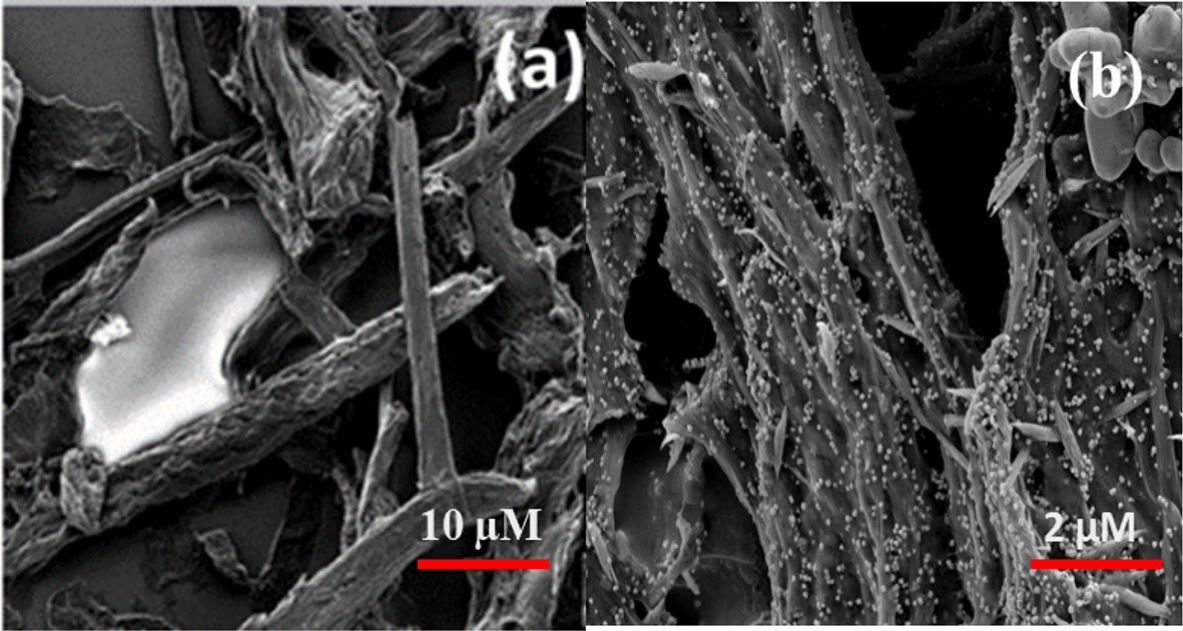
SEM micrographs of (**a**) CNF and (**b**) AgNPs@CNF.

Figure 11 depicts SEM micrographs of CNF and AgNPs@CNF composites.

**Figure 12:**
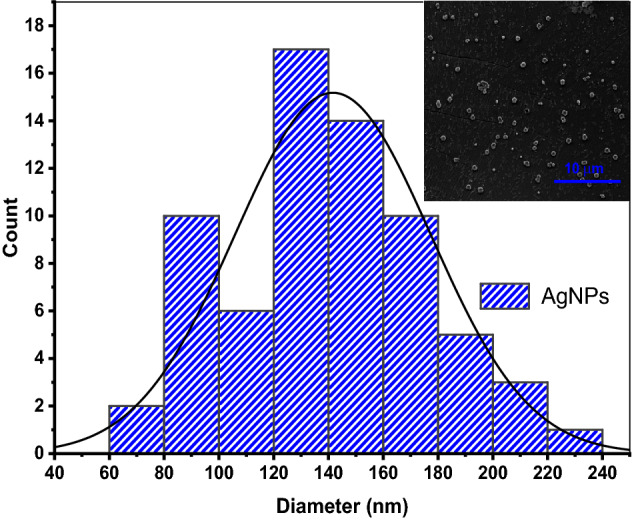
Size distribution of AgNPs embedded within PVA-CNF composite. Inset SEM micrographs of AgNPs@PVA-CNF.

From Fig. 11, it can be observed that cellulose was composed of rigid smooth fibers entangled amongst themselves without presence of AgNPs. In situ reduction of AgNPs could be observed in fibers afterwards as the fiber surface was decorated with small spherical AgNPs within the fibril network. It should be noted that celluloe nanofibrils agglomerate upon drying hence the absence of isolated fibrils with smaller sizes. However, the sizes of CNF from bagasse have been reported in a different study which also reported on the presence of AgNPs^41^. From Fig. 12, presence of AgNPs was confirmed from the SEM micrographs of nanocomposite consisting of AgNPs, CNF and PVA in which the sizes of AgNPs embedded within the composite network was found to be between 60 and 240 nm.

### Antimicrobial activity of composite films

**Figure 13:**
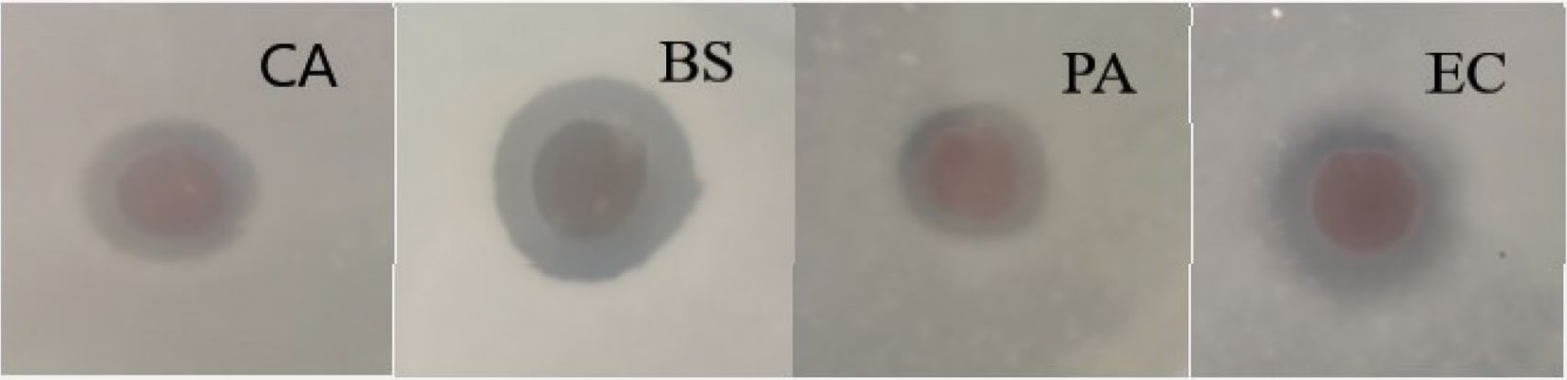
Inhibition of nanocomposite against *C. albicans* (Ca), *B. subtili* (Bs), and *P. aeruginosa* (Pa)*, E. coli* (Ec).

**Figure 14:**
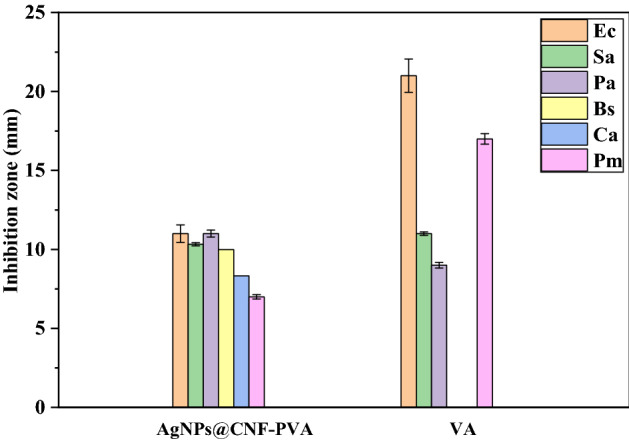
Inhibition zone assay of pure AgNPs@CNF-PVA films and vancomycin on the growth of *C. albicans* (Ca), *E. coli* (Ec)*, P. aeruginosa* (Pa)*, B. subtili* (Bs) and *P. mirabilis* (Pm).

The presence of the inhibition zone was used to assess the antimicrobial effect of the film towards the different strains of bacteria and the results are depicted in Figs. 13,14.

From Figs. 13,14, pure PVA film did not show any antimicrobial activity, since there was no inhibition zone recorded. In comparison, the AgNPs@CNF-PVA composite film formed strong inhibition zones around the disk as shown in Fig. 9, thus it can be considered to have antimicrobial abilities. By comparing these results with that of known standards as depicted in Fig. 10, Vancomycin had inhibitoty effects against all the bacteria used in this study apart from *C. albicans* and *B. subtili* (Bs). The AgNPs@CNF-PVA composite film ensures some degree of inhibition across all the tested strains, unlike the standards. The antimicrobial efficacy was evaluated through the analysis of known antibacterial standards which showed limited toxicity towards certain bacteria and demonstrated enhanced antimicrobial effects towards others as shown in Fig. 14. Silver exhibits strong bactericidal properties for a wide spectrum of bacteria, viruses and fungi^71,81,82^. AgNPs can attach themselves onto the bacterial membrane forming clusters that consistently tamper with the integrity of the cell wall to cause membrane damage, leading to bacterial death^83,84^. Metallic silver in itself is inert but it can react with moisture from the atmosphere to give silver ions^85^ which are highly toxic to most bacteria^86,87^. With these antibacterial test results, the hybrid AgNPs@CNF-PVA composite film has the potential for antibacterial packaging material.

## Conclusion

Hybrid antimicrobial AgNPs@CNF-PVA composite films were prepared and the applicability to function as a food packaging material was determined through measurement of their water vapour permeability (WVP), wettability (W) and solubility (S). The films were found to have excellent mechanical properties given their high tensile strength, Young’s modulus and their percent elongation. The composite films were found to have good antimicrobial properties as they were able to inhibit the growth of both gram-positive, gram-negative bacteria and a fungus used in this study. This inhibition was attributed to the presence of silver nanoparticles which have been reported to have antimicrobial properties against various microorganisms as in the case of this study. As such, with further modifications and blending with other polymers, this composite material can be an excellent replacement for petroleum-based packaging material which have been reported to be environmental contaminants.

## Data Availability

The datasets used and/or analysed during the current study available from the corresponding author on reasonable request.
